# Bipolar-barrier tunnel heterostructures for high-sensitivity mid-wave infrared photodetection

**DOI:** 10.1038/s41377-025-01905-y

**Published:** 2025-07-21

**Authors:** Fakun Wang, Song Zhu, Wenduo Chen, Ruihuan Duan, Tengfei Dai, Hui Ma, Congliao Yan, Shi Fang, Jianbo Yu, Yue Zhang, Qikan Dong, Wenjie Deng, Zheng Liu, Qi Jie Wang

**Affiliations:** 1 School of Electrical & Electronic Engineering, Nanyang Technological University, Singapore, 639798 Singapore; 2 School of Materials Science and Engineering, Nanyang Technological University, Singapore, 639798 Singapore; 3 Key Laboratory of Optoelectronics Technology, Ministry of Education, School of Information Science and Technology, Beijing University of Technology, 100124 Beijing, China; 4 Centre for Disruptive Photonic Technologies, School of Physical and Mathematical Sciences, Nanyang Technological University, Singapore, 637371 Singapore

**Keywords:** Mid-infrared photonics, Optical materials and structures

## Abstract

The rapid development of modern infrared optoelectronic technology has driven a growing demand for high-sensitivity mid-wave infrared (MWIR) photodetectors capable of reliable room-temperature operation. Achieving optimal specific detectivity, a critical performance metric for MWIR photodetection, remains challenging due to inherent limitations imposed such as high dark current, low optical absorption, or both. To address these challenges, we present an approach based on a bipolar-barrier architecture featuring a black phosphorus (BP)/MoTe_2_/BP tunnel heterostructure integrated with an Au reflector. This configuration delivers simultaneous electrical and optical enhancement, effectively suppressing dark currents and significantly increasing optical absorption. The bipolar-barrier structure minimizes dark current by blocking thermally excited and bias-induced carrier leakage, while facilitating efficient tunneling of photogenerated carriers via trap-assisted photogating mechanisms. In addition, the Au reflector enhances optical absorption through interference effects. As a result, the heterostructure achieves remarkable performance metrics, including a room-temperature specific detectivity of ∼3.0 × 10^10 ^cm Hz^0.5^ W^−1^, a high responsivity of ∼4 A W^−1^, and an external quantum efficiency of ∼140% within the MWIR range. These results establish the bipolar-barrier tunnel heterostructure as a highly efficient platform, paving the way for the next generation of advanced infrared optoelectronic devices.

## Abstract (web summary)

A bipolar tunnel heterostructure design is demonstrated that effectively suppresses dark current while facilitating the tunneling of photogenerated carriers, enabling high-sensitivity mid-infrared photodetection.

## Graphical abstract

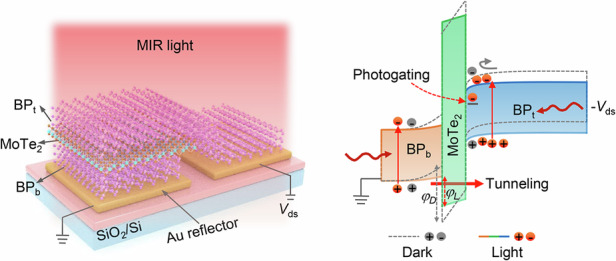

## Introduction

Mid-wave infrared (MWIR, 3–5 µm) photodetectors are essential for modern optoelectronic technology applications including night vision, medical diagnostics, optical communications, spectroscopy, and object inspection^1–8^. High-performance MWIR photodetectors with weak-light detection capabilities are particularly desirable. Specific detectivity (D*), a crucial performance metric, is strongly influenced by the suppression of dark current, which arises from mechanisms such as diffusion, generation-recombination (G-R) associated with Shockley-Read-Hall (SRH) processes, tunneling, and surface leakage current^9–11^. To overcome these limitations, various unipolar barrier structures (e.g. n-B-n and p-B-p) have been developed to suppress dark current by blocking majority carrier flow in conventional semiconductors (e.g. HgCdTe and III-V)^10,12,13^ and emerging two-dimensional (2D) materials (e.g. BP and b-AsP)^9,14,15^. Despite these advancements, achieving high performance at room temperature remains challenging due to thermally excited and bias-induced minority carriers that significantly contribute to dark current. Furthermore, the limited photon-trapping capability of nanoscale 2D materials constrains photoelectric conversion efficiency^16–22^. Therefore, simultaneous optimization of both electrical and optical properties is crucial for superior room-temperature MWIR photodetection.

In this work, we propose a bipolar-barrier structure based on 2D BP/MoTe_2_/BP heterostructures integrated with a Au reflector to achieve high-sensitivity MWIR photodetection at room temperature. In this architecture, the MoTe_2_ bipolar-barrier layer effectively blocks both majority holes and minority electrons from the p-type BP photoactive layers due to the intentionally introduced dual barrier in both the valence and conduction bands. This approach prevents dark current arising from the leakage of minority carriers, a key limitation of unipolar barrier structures. Furthermore, the inclusive of a Au reflector enhances optical absorption through interference effects. This design significantly suppresses dark current while enabling efficient tunneling of photogenerated hole carriers via trap-assisted photogating effects. As a result, the heterostructure exhibits outstanding performance metrics, as evaluated by the established characterization guidelines^23,24^, including a high room-temperature specific detectivity of ∼3.0 × 10^10 ^cm Hz^0.5^ W^–1^, a high responsivity of ∼4 A W^–1^, and an external quantum efficiency (EQE) of ∼140% in the MWIR region. These results highlight the promise of the bipolar-barrier tunnel heterostructure as an effective platform for high performance MWIR photodetection, offering new opportunities for advancement of next-generation infrared optoelectronic devices.

## Results

### Device design and optical optimization

**Fig. 1:**
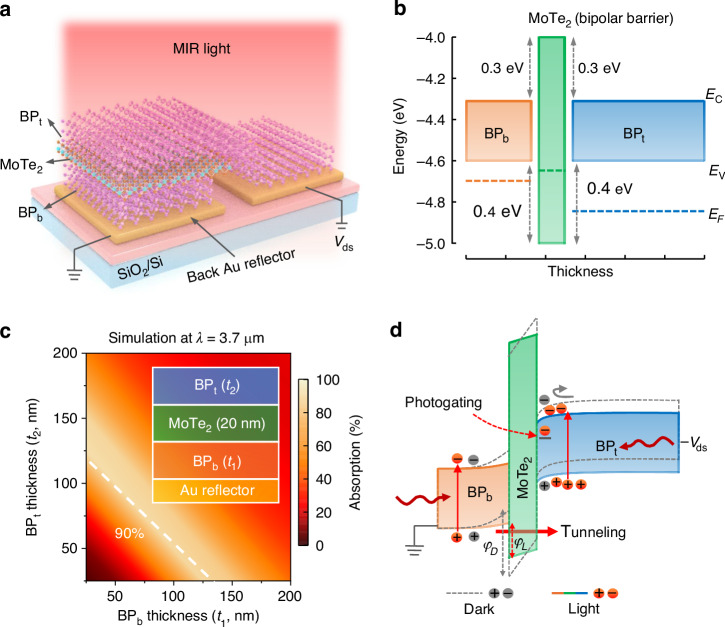
Bipolar-barrier tunnel heterostructures design and device optimization. **a** Schematic diagram of the BP_t_/MoTe_2_/BP_b_ bipolar-barrier tunnel heterostructure with a Au reflector. BP_t_, top black phosphorus; BP_b_, bottom black phosphorus. **b** Band profiles of BP_t_, MoTe_2_, BP_b_ before contact. **c** Simulated absorption for the BP_t_/MoTe_2_/BP_b_ heterostructures on a 50 nm Au reflector, as a function of the BP layer thickness. The thickness of MoTe_2_ is set to 20 nm. **d** Band alignment of the heterostructure under a negative bias and mid-wave light illumination. Dark carriers and photogenerated carriers in BP are marked in gray and red, respectively. Under mid-infrared light illumination, the effective barrier height in the valence bands of BP/MoTe_2_ heterostructure decreases compared to the dark condition, promoting quantum tunneling of photo hole carriers and resulting in significant tunneling photocurrents. And the photogating effect induced by trap states in BP modulates carriers transport dynamics, contrbuting to gain. *φ*_D_ and *φ*_L_ refer to the effective barrier height in the dark and under light illumination, respectively

The specific detectivity is a key figure of merit for infrared detectors, often limited by high dark current and low responsivity. Optimizing this detectivity requires simultaneous suppression of dark current and enhancement of optical absorption. Figure 1a shows the proposed bipolar-barrier design based on a BP/MoTe_2_/BP heterostructure stacked on a Au reflector. In this structure, narrow-bandgap BP and wide-bandgap MoTe_2_ act as the MWIR photoactive layer and bipolar-barrier layer, respectively. The energy levels of BP and MoTe_2_, shown in Fig. 1b, were obtained from previous works^25^ and Kelvin probe force microscopy (KPFM) characterizations (Fig. S1). Specifically, a type-I band alignment is formed in the BP/MoTe_2_ heterostructure, where both the valence and conduction bands exhibit band offsets. The conduction band offset of ∼0.3 eV blocks minority electrons generated by thermal excitation and external bias in the p-type BP layers. Meanwhile, the valence band offset of ∼0.4 eV blocks some majority holes, while still allowing a portion of the holes to cross the barrier through tunneling under bias. This dual-blocking mechanism eliminates minority carrier leakage, thereby reducing dark current compared to unipolar structures (Fig. S2). The photogenerated current is conducted through a tunneling process, which is also suppressed by MoTe_2_ barrier layer. However the tunneling process of the photo carriers is much more efficient than that of dark carriers due to the reduced effective barrier heights between MoTe_2_ and BP at the valence band edge caused by trap-assisted photogating effects under illumination (Fig. 1d). More detailed explanations are provided in the following sections.

The optical absorption is enhanced due to the constructive interference between the reflected light from the Au reflector and the incident light. To obtain an optimal MWIR absorption efficiency, we simulated the light absorption of the heterostructure in the wavelength range of 2–5 μm based on the reported refractive index^26^ of BP using finite-difference time-domain (FDTD) solutions. BP features an anistropic crystalline structure, enabling a higher absorption in its armchair (AC) direction (Fig. S3). All simulated results were obtaind by setting the light polarization parallel to its AC direction. As provided in Fig. S4a, b, the heterostructure with a Au reflector exhibits strong absorption enhancement within the wavelength range of 2-4 μm with a maxunium enhancement factor at λ ∼3.7 μm. Figure 1c demonstrates the simulated absorption of the heterostructure on a Au reflector along with the AC direction of BP at λ = 3.7 μm. The absorption exhibits a distinct periodicity dependent on the thickness of the bottom BP (BP_b_) and the top BP (BP_t_), which should be ascribed to variations in front surface reflection resulting from thickness-dependent constructive and destructive interference of MWIR light^26^. From this result, the maximum absorption (∼90%) occurs where the sum of the thickness of BP_t_ and BP_b_ is about 150 nm. Furthermore, the absorption as a function of MoTe_2_ barrier layer thickness is simulated and presented in Fig. S4c, d, suggesting the optimal MoTe_2_ thickness is around 20-30 nm.

Since optimizing both optical absorption and dark current suppression is crucial for achieving high-performance photodetection, we systematically characterized the photoresponse of the heterostructures by varying the thicknesses of BP and MoTe₂ (Figs. S5–10). Our results indicate that the dark current exhibits an approximately exponential increase with increasing MoTe_2_ thickness (Figs. S5–6). This behavior can be attributed to a significant reduction in the surface potential difference between MoTe_2_ and BP as the MoTe_2_ thickness increases (Fig. S1). A lower surface potential difference leads to a weaker barrier effect, making it easier for the majority carriers (holes) in BP to overcome the energy barrier, thereby resulting in higher dark current levels. Based on the optical absorption simulations, a 20 nm-thick MoTe_2_ was selected as the optimal bipolar-barrier layer. BP thickness selection must also consider its environmental stability and thickness-dependent optical bandgap. Thinner BP layers (below ~50 nm) tend to degrade more rapidly due to oxidation and ambient exposure, whereas excessively thick BP layers increase dark current due to enhanced majority carrier transport (Figs. S7–10). Based on these considerations, we determined that the optimal thicknesses are 100 nm for the top BP (BP_t_), 50 nm for the bottom BP (BP_b_), and 20 nm for the MoTe_2_ barrier layer. These thicknesses not only ensure strong optical absorption but also effectively suppress dark current, leading to improved device performance in terms of high responsivity and specific detectivity. Leveraging the optical design, the light absorption of a typical device with BP_t_/MoTe_2_/BP_b_ (∼103/22/45 nm) on Au (50 nm) reaches up to ~75% along with the AC direction at λ = 3.7 μm. The thickness and crystalline orientation of the fabricated heterostructure are presented in Fig. S11, which were determined through atomic force microscopy (AFM) and polarized Raman spectroscopy, respectively.

### Dark current generation and transport mechanism

**Fig. 2:**
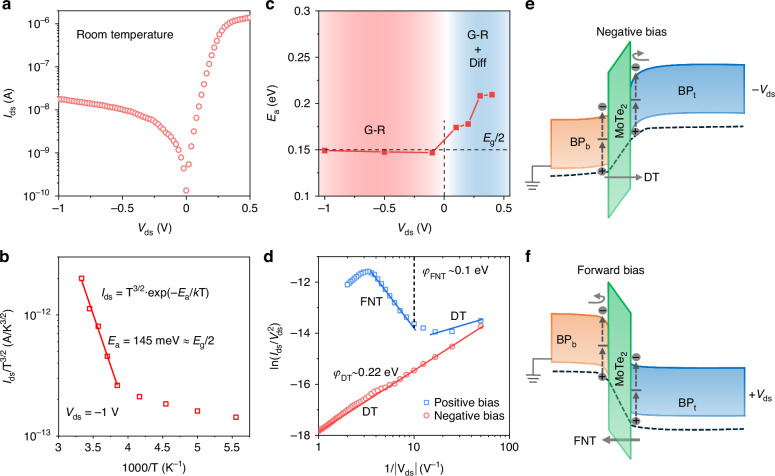
Dark current mechanism of the bipolar-barrier heterostructure. **a** *I*_ds_-*V*_ds_ curve of the BP_t_/MoTe_2_/BP_b_ ( ∼ 103/22/45 nm) heterostructure in the dark and room temperature. **b** Dark current as a function of temperature at *V*_ds_ = -1 V. The data was fitted in the high temperature region using the Arrhenius plot with an activation energy (*E*_*a*_) of ∼ 145 meV. **c** Activation energy at different biases. **d** ln(*I*/*V*^2^) verse 1/ | *V*| plots for negative and positive bias, which corresponds to the dark current shown in (**a**). The negative bias region shows directing tunneling (DT) behavior with a barrier height of 0.22 eV. For the positive bias region, directing tunneling and Fowler-Nordheim tunneling (FNT) were observed at low and high biases, respectively. The barrier height for FNT is evaluated to ∼ 0.1 eV. Band diagrams of the tunneling heterostructure at (**e**) negative and (**f**) positive bias. Under negative bias, electrons in the BP_t_ region are blocked by the conduction band barrier, while holes in the BP_b_ region can tunnel directly through the valence band barrier. Conversely, under positive bias, electrons in the BP_b_ region are impeded by the conduction band barrier, whereas holes in the BP_t_ region traverse the valence band barrier via direct tunneling and Fowler–Nordheim tunneling

The room-temperature *I*_ds_-*V*_ds_ characteristics of the BP_t_/MoTe_2_/BP_b_ (∼103/22/45 nm) heterostructure in the dark are shown in Fig. 2a. The dark current of the bipolar-barrier BP_t_/MoTe_2_/BP_b_ heterostructure at negative bias is several orders of magnitude lower than that of non-barrier BP_t_/BP_b_ (∼94/56 nm) heterostructure (Fig. S12), and is also lower than that of previously reported unipolar-barrier heterostructures (BP/MoS_2_/Gr, b-AsP/MoS_2_/BP)^9,14,27^ and BP/MoS_2_ p-n heterostructure^13^, confirming the effective blocking of dark current in the bipolar-barrier heterostructure.

The dark current generation mechanism in the heterostructure was analyzed using Arrhenius plots of the dark current versus temperature. As shown in Fig. 2b and Fig. S13, the dark current is well described by the relation *I*_ds_ ∼ T^3/2^exp(-*E*_a_/*k*T) in the high-temperature regime (260-300 K), where *E*_a_ and *k* refer to the activation energy and Boltzmann constant, respectively. The diffusion current typically follows a temperature dependence of the form T^3^exp(-*E*_g_/*k*T), while the generation-recombination (G-R) current scales as T^3/2^exp(-*E*_g_/2*k*T), where *E*_g_ is the bandgap of the active layer^10^. This implies that the effective activation energy *E*_a_ for diffusion current is approximately *E*_g_, whereas for G-R current it is about *E*_g_/2. Under negative bias, the measured activation energy is around 0.15 eV (Fig. 2c), which is very close to half of the BP bandgap (~0.3 eV). This observation suggests that, above 260 K, the dark current is predominantly governed by the G-R mechanism via the Shockley–Read–Hall (SRH) process involving midgap traps^28^. These traps are primarily induced by surface oxidation (PO_x_) of BP, as confirmed by AFM and Raman characterizations (Fig. S8). In contrast, the activation energy at positive bias (*E*_a_ = 0.17–0.21 eV) is slightly higher than half of the BP bandgap, indicating that while the G-R process still plays a significant role, there is an additional contribution from diffusion current^13^. Although the positive bias lowers the barrier height, allowing more carriers to overcome it and contribute to the dark current, the number of carriers generated solely through the G-R mechanism is insufficient to account for the observed current levels. An extra supply of carriers is thus provided by direct valence-to-conduction band transitions, which require energy nearly equal to the full bandgap of BP. This additional energy requirement explains the higher activation energy observed under positive bias.

In our designed BP/MoTe_2_/BP bipolar barrier heterostructure, the employed BP is heavily p-doped, resulting in a significantly higher hole concentration compared to electron concentration. The MoTe₂ barrier layer plays a crucial role in influencing the transport properties. Specifically, the electron barrier height in MoTe_2_ is approximately 0.3 eV, which is notably higher than the barrier for hole transport. As a result, the electron transport is more strongly hindered by the MoTe_2_ barrier compared to hole transport. Carrier transport across the MoTe_2_ barrier primarily occurs through two mechanisms: thermionic emission and quantum tunneling. To assess which mechanism is more dominant, we analyzed the current-voltage characteristics under bias, using both thermionic emission and tunneling models.

According to the thermionic emission theory, the current density through the barrier can be determined by the formal^29^:

1 $$J={J}_{0}\exp \left(\frac{qV}{nkT}\right)\left[1-\exp \left(-\frac{qV}{kT}\right)\right]$$

2 $${J}_{0}={A}^{\ast }{T}^{2}\exp \left(-\frac{q\varphi }{kT}\right)$$

where A* is the Richardson constant, T is temperature, *k* is the Boltzmann constant, *q* is the electronic charge, *V* is the bias voltage, and *n* is the ideality factor. The ideality factor *n* can be extracted by $n=\frac{q}{{kT}}\frac{{dV}}{d(\mathrm{ln}J)}$. As shown in Fig. S14, the ideality factor *n* is significantly greater than 1 under both negative and positive bias conditions, indicating a discrepancy between the thermionic emission model predictions and the experimental observations.

Furthermore, we analyzed the transport characteristics using tunneling models, including directing tunneling (DT) and Fowler-Nordheim tunneling (FNT)^30–32^:

3 $${I}_{DT}\propto Vexp\left(-\frac{4\pi d\sqrt{{m}^{\ast }\varphi }}{h}\right)$$

4 $${I}_{FNT}\propto {V}^{2}\exp \left(-\frac{8\pi d\sqrt{2{m}^{\ast }{\varphi }^{3}}}{3hqV}\right)$$

where *d*, *φ*, m*, *q*, and *h* is the barrier width, barrier height, effective mass, elementary charge, and the Plank constant, respectively. By analyzing the measured *I*_ds_-*V*_ds_ characteristics, we found that the tunneling model accounts more for the carrier transport behaviors of the bipolar-barrier BP_t_/MoTe_2_/BP_b_ heterostructure, as demonstrated in Fig. 2d and Fig. S15. The plot of ln(*I*/*V*^2^) vs. 1/*V* shows a logarithmic rise in the negative bias region, indicating a well fitting of the DT model. The DT barrier height (*φ*_DT_) was evaluated to be ∼0.22 eV according to the intercept $\frac{4\pi d\sqrt{{m}^{* }\varphi }}{h}$, which is close to the difference between the Fermi level of BP_b_ and the maximum valence band of MoTe_2_. For the positive bias, the fitting of the ln(*I*/*V*²) vs. 1/*V* curve reveals a logarithmic decay for FNT at high bias, while it shows a logarithmic increase for DT at low bias. The transition from DT to FNT at the bias voltage *V*_DT-FNT_ can be determined from the crossover point between the two regions^30^. This point corresponds to the maximum barrier height for FNT (*φ*_FNT_ = e*V*_DT-FNT_ ∼0.1 eV), which is approximately equal to the difference between the Fermi level of BP_t_ and the maximum valence band of MoTe₂.

Based on the above analysis, the carrier transport in this heterostructure is better described by the tunneling model. The tunneling probability for carriers can be approximated by the formula: $T(E)\propto \exp (-2d\sqrt{2{m}^{* }\varphi }/{\rm{\hslash }})$, where $\varphi$ and $d$ are the barrier height and width, respectively. In our structure, the higher barrier height for electrons makes tunneling through the MoTe_2_ layer highly improbable. Therefore, MoTe_2_ effectively functions as an electron-blocking barrier, significantly suppressing electron transport while allowing easier hole transport via tunneling. The energy band diagrams in Fig. 2e, f illustrate the transport processes in the bipolar-barrier BP_t_/MoTe_2_/BP_b_ tunnel heterostructure under different bias conditions.

### Room-temperature mid-infrared photodetection performance

**Fig. 3:**
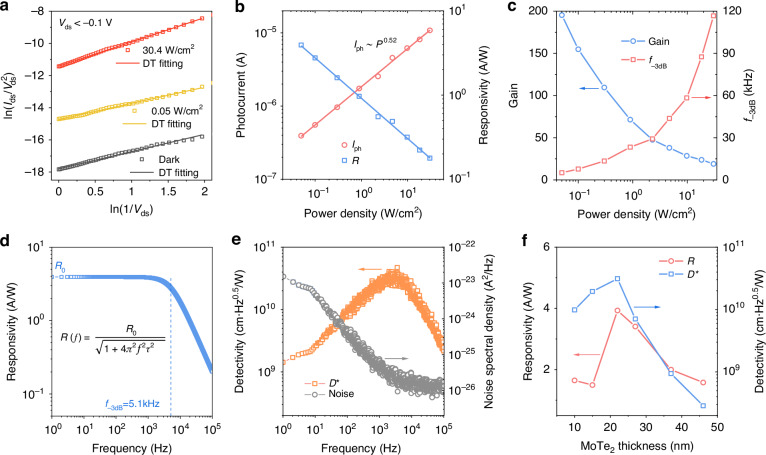
Photoresponse characterizations of the bipolar-barrier tunnel heterostructures. **a** Directing tunneling (DT) plots of the heterostructure in the negative bias regime in the dark and under a 3.7 μm laser illumination. The linear relationship between ln(*I*/*V*^2^) and ln(*I*/*V*) indicates the direct tunneling behaviors of the heterostructure in the dark and under illumination, respectively. **b** Photocurrent and responsivity as a function of power density at *V*_ds_ = –1 V. **c** Gain and –3dB bandwidth as a function of power density at *V*_ds_ = –1 V. **d** Frequency dependence of responsivity at *V*_ds_ = –1 V. The responsivity is calculated at the power density of 0.05 W cm^–2^ with a -3dB bandwidth of *f*_-3dB_ = 5.1 kHz. **e** Frequency-dependent noise spectral density and specific detectivity at the power density of 0.05 W cm^–2^ and *V*_ds_ = –1 V. **f** Responsivity and detectivity as a function of MoTe_2_ thickness

Under MWIR light (λ = 3.7 μm) illumination with various power densities, the bipolar-barrier BP_t_/MoTe_2_/BP_b_ heterostructure generated significant photocurrents, as shown in Figs. S16–17. This strong photoresponse can be attributed to enhanced light absorption due to the Au back reflector. Additionally, tunneling-dominated carrier transport contributes to the excellent photoresponse (Fig. S16b, c). The photodetection performance of the heterostructure was primarily investigated under negative bias due to its lower dark current. As described in Fig. 3a, the good linear relationship between ln(*I*/*V*^2^) and ln(*I*/*V*) at different power densities indicates that the heterostructure’s photoresponse is dominated by DT under negative bias, with the DT barrier height decreasing as power density increases (Fig. S16d). This results in higher photocurrents at higher power densities.

Figure 3b illustrates the photocurrent sub-linearly increases as the increment of power density (*I*_ph_ *∼ P*^0.52^) at *V*_ds_ = –1 V, suggesting the presence of trap states in the heterostructure^29,33^. Based on AFM and Raman characterizations (Fig. S8), we believe that trap states mainly originate from phosphorus oxides (PO_x_) on the surface of BP. The resulting PO_x_ species introduce trap states at the BP/MoTe_2_ interface, generating a localized electric field. Under mid-infrared laser illumination, this field effectively captures photogenerated electrons within the BP, thereby functioning as a local gate to modulate the transport of photogenerated carriers via the photogating effect. The impact of this local gate on carrier transport properties results in the decrease of responsivity with increasing the light intensity (Fig. 3b). This behavior can be attributed to the saturation of trap states at higher intensities, which reduces the efficacy of the photogating effect. Accordingly, the photoresponse of the heterostructure under negative bias is illustrated in the energy band diagram shown in Fig. 1d. The photogenerated holes in BP_b_ undergo direct tunneling into BP_t_, where they, along with the photogenerated holes generated in BP_t_, are collected by the negative electrode. At the same time, a portion of the photogenerated electrons in BP_b_ are trapped by localized states, while the rest are extracted by the positive electrode. Similarly, in BP_t_, some photogenerated electrons are captured by localized states, while others are blocked by the barrier, preventing their collection.

The traps-induced photogating effect introduced photoconductive gain (G) in the heterstructures, which can be calculated using the quation *G* = τ_0_/τ_trans_, where τ_0_ and τ_trans_ represent the minority carrier recombination lifetime and carrier transit time, respectively. The recombination lifetime τ_0_ of photogenerated minority carriers is considered equivalent to the photoresponse decay time. As shown in Fig. S18, the recombination process of photo-generated electrons and holes becomes faster under a higher light intensity for both 1 V and –1 V bias. This behaviour can be attributed to the increased generation of photo-generated carriers at higher light intensities, which progressively fills the trap states at the heterostructure interface. As the traps are filled, the likelihood of capturing photo-generated electrons decreases, leading to faster electron-hole recombination and thus a faster response time. At the same light intensity, the response time is slightly faster at a 1 V bias compared to −1 V, which can be attributed to the lower tunneling barrier for carriers under positive bias. This reduced barrier enables quicker carrier transport, enhancing the overall recombination rate and leading to a faster response time. The transit time τ_trans_ is estimated according to the formula τ_trans_ = *L*^2^/*μV*_ds_, where *L* is the transit distance and *μ* is the carrier mobility. More details can be found in Fig. S19. The deduced gain (*G*) reaches up to 195 at a low power level of 0.05 W cm^–2^ (Fig. 3c), but decreases as the incident power density increases, due to the limited number of localized trap states^34^. Additionally, the -3dB photoresponse bandwidths at different power densities were calculated using *f*_-3dB_ = 0.35/τ_rising_^35^, where τ_rising_ the photoresponse rising time (Fig. S18). As displayed in Fig. 3c, the *f*_-3dB_ increases from ∼5.1 to ∼116 kHz as the incident power density rises from 0.05 to 30.4 W cm^-2^ at *V*_ds_ = −1 V, which is attributed to faster free carriers recomnination due to the trap saturation under higher photon injection conditions.

The key figures of merits such as responsivity (*R*) and specific detectivity (D*) are studied here. The *R* can be deduced using *R* = *I*_ph_/(*P* × A), where *I*_ph_, *P*, and A refer to the photocurrent, incident power density, and effective device area. The effective device area of the heterostructure is about 200 μm^2^. It should be noted that the responsivity shown in Fig. 3b is calculated with the device under constant irradiation, which is defined as *R*_0_. Due to the trapping-induced photogating effect, the *R*_0_ shows sublinear dependence on the incident power density, reaching up to ∼4 A W^–1^ at the power density of 0.05 W cm^–2^ (Fig. 3b). Correspondingly, the external quantum efficiency (EQE = *Rhc*/*q*λ) was calculated to be ∼140%. The D* can be obtained according to the formula D* = A^0.5^/NEP, where NEP refers to the noise equivalent power normalized to 1 Hz. The NEP is calculated following NEP = *i*_N_/*R*, where *i*_N_ is the noise current density. Figure 3e shows the noise characteristics of the heterostruature, which contains frequency-dependent 1/*f* and g-r noise and frequency-independent white noise (thermal noise and shot noise). In order to accurately estimate the specific detectivity, we evaluated the frequency-dependent responsivity according to the equation *R*(*f*) = *R*_0_/(1 + 4π^2^*f*^2^τ^2^)^0.5^, where *f* is the frequency and τ is the time constant (τ = 1/2π*f*_-3dB_)^24^. The evaluated responsivity as a function of frequency is presented in Fig. 3d. Consequently, the peak D* of the heterostructure is evaluated to be ∼3.0 × 10^10 ^cm Hz^0.5^ W^−1^ at a frequency of 3 kHz and a power density of 0.05 W cm^-2^, as displayed in Fig. 3e. This value is higher than that of previously reported unipolar barrier photodetectors based on 2D heterostructures^9,14,15,27^, as shown in Table 1. The critical breakthrough lies in synthesizing high-quality 2D materials with fewer intrinsic defects and improving the heterostructure interface quality through optimizing device fabrication processes^36–42^. Figure 3f illustrates the influence of MoTe_2_ thickness on device performance, showing an initial improvement followed by a decline as thickness increases. The optimal responsivity of ∼ 4 A W^−1^ and detectivity of ∼ 3.0 × 10^10 ^cm Hz^0.5^ W^–1^ were achieved at a MoTe_2_ thickness of approximately 20 nm. This trend results from a balance between carrier blocking and tunneling effects. When MoTe_2_ is too thick (>30 nm), the reduced valence band offset weakens the barrier, allowing more carriers to overcome it and increasing dark current, which degrades performance. Conversely, when MoTe_2_ is too thin (<20 nm), the tunneling probability rises significantly, also leading to higher dark current and reduced performance.

**Table 1:** Performance comparison of the bipolar-barrier design with unipolar barrier structures and commercial HgCdTe at room temperature

Device configuration		λ (μm)	R (A W^−1^)	D* (cm Hz^0.5^ W^−1^)	Response time	Ref.
HgCdTe (VL5T0)	PIN	4.75	1	1.5 × 10^10^	0.12 μs	Thorlabs
HgCdTe (PVI-4-1×1-TO39-NW-36)	PIN	3.5	1.8	6.0 × 10^10^	0.15 μs	VIGO
b-AsP/WS_2_/b-AsP (50/6/40 nm)	unipolar	4.6	6.47 × 10^−6^	2.64 × 10^7^	200 μs	15
BP/MoS_2_/Gr (47/14/5.5 nm)	unipolar	Blackbody @ 1173 K	-	2.3 × 10^10^	28/23 μs	9
BP/MoS_2_/b-AsP (11/3/31 nm)	unipolar	Blackbody @ 573 K	0.0234	2.7 × 10^10^	0.4/0.6 μs	14
BP/MoS_2_/b-AsP (300/20/32 nm)	unipolar	Blackbody @ 773 K	0.857	2.3 × 10^10^	0.5/0.5 μs (1550 nm)	27
BP/MoTe_2_/BP (103/22/45 nm)	bipolar	Blackbody @ 773 K	3	2.8 × 10^10^	78/103 μs	This work
		3.7	4	3.0 × 10^10^	3/8 μs	

### Blackbody response characteristics

**Fig. 4:**
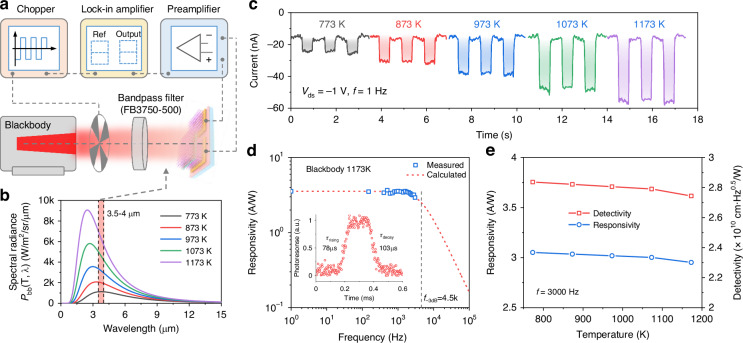
Blackbody response of the bipolar-barrier tunnel heterostructures. **a** Schematic of the blackbody response measurement setup. **b** Blackbody radiation spectra at different temperatures, which were calculated according to the Planck’s law. An optical bandpass filter is used to allow light within the wavelength range of 3.5–4 μm to pass through. **c** Time-resolved blackbody response of the heterostructure at different radiation temperatures. **d** Blackbody responsivity as a function of switching frequency at the temperature of 1173 K. Insert shows the blackbody response time of 78/103 μs, indicating a –3dB bandwidth of 4.5 kHz (*f*_-3dB_ = 0.35/τ_rising_). **e** Responsivity and specific detectivity at different blackbody temperatures

The practical application potential of the bipolar-barrier BP_t_/MoTe_2_/BP_b_ tunnel heterostructure was demonstrated through blackbody detection at room temperature. Figure 4a illustrates the schematic of blackbody response measurement setup, where an optical chopper was used to modulate the input switching frequency and a lock-in amplifier equipped with a preamplifier was used to record the output signal. To compare the heterostructure’s detection performance for blackbody radiation and laser radiation, we placed an optical band-pass filter with a central wavelength of 3.75 μm and a bandwidth of 0.5 μm in front of the device. This filter allows only mid-infrared radiation in the wavelength range of 3.5–4 μm to reach the heterostructure. The spectral radiance of the blackbody at various temperatures, ranging from 773 to 1173 K (Fig. 4b), was calculated based on the blackbody radiation law:

5 $${P}_{{bb}}\left(T,\lambda \right)=\frac{2h{c}^{2}}{{\lambda }^{5}}\frac{1}{{e}^{{hc}/(\lambda {k}_{b}T)}-1}$$

where *P*_bb_(T, λ) is the blackbody radiation power at temperature T and wavelength λ, *h* is the Planck constant, *c* is the speed of light, and *k*_B_ is the Boltzmann constant. As shown in Fig. 4c, the heterostructure exhibited a stable and repeatable photoresponse for different blackbody temperatures at a chopper frequency of 1 Hz and a bias of *V*_ds_ = −1 V. The blackbody response rising and decay time were evaluated to be 78 and 103 μs (Fig. 4d), respectively, corresponding to a *f*_-3dB_ of 4.5 kHz. The response bandwidth of the blackbody is slightly lower than that of the laser at a low power level of 0.05 W cm^−2^, which may be attributed to the non-monochromatic nature of the blackbody radiation spectrum reaching the heterostructure.

For blackbody detection, the blackbody responsivity (*R*_bb_) can be evaluated by *R*_bb_ = *I*_ph_/*P*_bb-d_, where *P*_bb-d_ represents the effective blackbody radiation power on to the device^27^. In our case, *P*_bb-d_ is given by *P*_bb-d_ = η*P*_bb_, where η represents the fraction of power passing through the band-pass filter, and *P*_bb_ is the power reaching the heterostructure surface without the filter. Both η and *P*_bb_ can be calculated using the following expressions:

6 $$\eta =\frac{\mathop{\int}\nolimits_{\lambda =3.5}^{\lambda =4}{P}_{{bb}}\left(T,\lambda \right)d\lambda }{\mathop{\int}\nolimits_{\lambda =0}^{\lambda =15}{P}_{{\rm{bb}}}\left(T,\lambda \right)d\lambda }$$

7 $${P}_{{bb}}\left(T\right)=\frac{\sigma {r}^{2}A}{{d}^{2}}$$

8 $$\sigma =\frac{2{\pi }^{5}{k}_{b}^{4}}{15{h}^{3}{c}^{2}}({T}^{4}-{T}_{0}^{4})$$

where σ is blackbody radiation rate that can be calculated by Stefan-Boltzmann law, T is the blackbody temperature, T_0_ is the background temperature (T_0_ = 300 K), r is aperture radius of the blackbody (r = 7.6 mm), A is the effective device area (A ∼ 200 μm^2^), and d is the distance between the aperture and the heterostructure (d = 10 cm). The calculation results were provided in Fig. S20_._ Accordingly, the frequency-dependent *R*_bb_ for a blackbody temperature of 1173 K was obtained, as shown in Fig. 4d, which is well matched with the calculated values using *R*(*f*) = *R*_0_/(1 + 4π^2^*f*^2^τ^2^)^0.5^ (τ = 1/2π*f*_-3dB_). Figure 4e presents the evaluated *R*_bb_ for blackbody temperatures ranging from 773 to 1173 K, with a value of ∼ 3 A W^−1^ at a frequency of 3 kHz. Correspondingly, the blackbody specific detectivity (*D*_bb_*) is estimated by *D*_bb_*= *R*_bb_A^0.5^/*i*_N_, which is approach to ∼ 2.8 × 10^10 ^cm Hz^0.5^ W^−1^ (Fig. 4e), which is competitive with that of previous works^9,14,26,27^. Overall, the detection performance of the heterostructure for blackbody radiation is similar to that for laser radiation. The slight differences may be due to the non-monochromatic and non-collimated nature of blackbody radiation.

## Discussion

In summary, we have designed and demonstrated a bipolar-barrier BP/MoTe_2_/BP tunnel heterostructure stacked on a Au reflector for high-sensitive mid-infrared photodetection. In the heterostructure, the dark current can be significantly suppressed by the bipolar barrier and the photocarriers are able to effectively tunnel the barrier. Moreover, a high optical absorption of 75% is achieved due to the interference of BP/MoTe_2_/BP stacked on a Au back reflector. Attributed to the synergistic effect of dark current reduction and optical absorption enhancement as well as photocurrent tunneling, the heterostructure shows an impressive room-temperature specific detectivity of ∼ 3.0 × 10^10 ^cm Hz^0.5^ W^−1^ with a high responsivity of ∼ 4 A W^–1^ as well as an EQE of 140% in the MWIR range.

In our proposed bipolar-barrier design, careful tuning of the barrier height is crucial. A smaller valence band offset facilitates the transport of majority holes across the barrier, which can diminish the photocurrent’s blocking effect. However, this also increases the dark current, potentially yielding higher responsivity at the expense of specific detectivity. Therefore, optimizing the band alignment is essential to achieve a balance between suppressing dark current and maintaining efficient photocurrent extraction.

Moreover, by harnessing the diverse range of available 2D materials and their adjustable band structures, the bipolar-barrier architecture can be tailored to operate across various wavelength ranges, including the visible, near-infrared, and long-wave infrared regions. Additionally, combining 2D materials with different bandgaps as photoactive layers could enable bias-controlled multi-band detection or broadband spectral reconstruction, further enhancing the versatility of this approach.

## Materials and methods

### Optical absorption simulation

Optical absorption simulations of the Au/BP_b_/MoTe_2_/BP_t_ heterostructures were performed using finite-difference time-domain (FDTD) solutions. The optical constants of BP and MoTe_2_ were taken from previous works^26,43^, while the optical properties of Au in the MWIR region were described using the Drude model. The Au layer thickness was fixed at 50 nm. A broadband (2–5 μm) linearly polarized plane wave was used as the light source, with the polarization direction aligned parallel to the armchair crystalline orientation of BP, as BP exhibits anisotropic optical properties with maximum absorption along this direction. Perfectly matched layers (PML) were applied as boundary conditions in the propagation direction to eliminate artificial reflections, while periodic boundary conditions were set perpendicular to the propagation direction to simulate an infinite array of unit cells. To quantify the total absorption, a reflection monitor was placed above the light source to measure the reflectance (Ref), and a transmission monitor was positioned beneath the Au layer. Since Au is optically opaque in this wavelength range, the transmittance (Trans) was set to zero. The total absorption (Abs) of the heterostructure was then calculated as (Abs = 1 – Ref). By extracting the reflectance from the reflection monitor, the total absorption efficiency of the device was obtained. Additionally, to optimize absorption efficiency, a parametric sweep over the thicknesses of BP_b_, MoTe_2_, and BP_t_ was performed. This enabled us to determine the optimal layer thicknesses that maximize light absorption.

### Device fabrication and characterization

The heterostructures demonstrated in this work were fabricated using a PDMS-assisted dry transfer technique. The as-used 2D BP and MoTe_2_ flakes were obtained by mechanically exfoliating bulk crystals purchased from HG graphene. The freshly exfoliated 2D BP and MoTe_2_ flakes were transferred onto the pre-prepared Au pads in BP_b_/MoTe_2_/BP_t_ order. The Au pad with a thickness of 50 nm acts as both a contact electrode and a mid-infrared back reflector. In addition, the transfer process prevents 2D flakes in the electrode contact interface area from being damaged by high-energy metal deposition, which could reduce the contact barrier to some extent^44^. The as-fabricated Au/BP_b_/MoTe_2_/BP_t_ heterostructures were encapsulated by a h-BN layer and finally annealed in a vacuum at 250 °C for 2 h. The crystalline orientations of the BP flakes were determined by polarized Raman spectroscopy (alpha-300R, WITec). Mid-infrared absorption spectra were obtained using an FTIR microscope (Vertex 80 v, Bruker). The thicknesses of fabricated BP_b_/MoTe_2_/BP_t_ heterostructures were determined by atomic force microscopy (NX10, Park).

### Electrical and optoelectrical measurements

The electrical properties of the BP_b_/MoTe_2_/BP_t_ heterostructures were conducted using a semiconductor parameter analyzer (B2912A, Keysight). A wavelength-tunable mid-infrared quantum cascade laser (Daylight Solution, MWIRCat) was used to investigate the optoelectrical properties of the heterostructures. A zinc selenide lens with a focal length of 7.5 cm was used to focus the laser. The speckle of the focused laser beam is about 100 μm in diameter, which can uniformly cover the entire BP_b_/MoTe_2_/BP_t_ heterostructures. The incident power was measured by a thermal power meter (Nova display-ROHS, OPHIR). The response time of the heterostructure was collected employing an oscilloscope (DSOX3054T, Keysight). The noise spectral density was obtained using a signal analyzer (N9030B, Keysight). The blackbody radiation was obtained from a blackbody source (Model 480, Gooch & Housego), which was modulated by a mechanical chopper. The photocurrent singal was recorded by a lock-in amplifier (SR830, Stanford Research Systems) equipped with a preamplifier (SR570, Stanford Research Systems). All optoelectrical measurements were carried out under ambient conditions at room temperature.

## Supplementary information

Supplementary Information

## Data Availability

The data that support the findings of this study are available from the corresponding author upon reasonable request.
