# Supplementary material for: Bipolar-barrier tunnel heterostructures for high-sensitivity mid-wave infrared photodetection
Source: Light Sci Appl. 2025 Jul 21;14:246. doi: 10.1038/s41377-025-01905-y (PMC12279951; doi:10.1038/s41377-025-01905-y)
Supplement: Supplementary file 1 — Supplementary Information [file 41377_2025_1905_MOESM1_ESM.docx]

Supplementary Information for

Bipolar-barrier tunnel heterostructures for high-sensitivity mid-wave infrared photodetection

Fakun Wang,^1^ Song Zhu,^1^ Wenduo Chen,^1^ Ruihuan Duan,^2^ Tengfei Dai,^1^ Hui Ma,^1^ Congliao Yan,^1^ Shi Fang,^1^ Jianbo Yu,^1^ Yue Zhang,^1^ Qikan Dong^1^, Wenjie Deng,^3^ Zheng Liu,^2^ and Qi Jie Wang^1,4^*

^1^School of Electrical & Electronic Engineering, Nanyang Technological University, Singapore 639798, Singapore.

^2^School of Materials Science and Engineering, Nanyang Technological University, Singapore 639798, Singapore.

^3^Key Laboratory of Optoelectronics Technology, Ministry of Education, School of Information Science and Technology, Beijing University of Technology, Beijing 100124, China

^4^Centre for Disruptive Photonic Technologies, School of Physical and Mathematical Sciences, Nanyang Technological University, Singapore 637371, Singapore.

*Corresponding author. Email: [qjwang@ntu.edu.sg](mailto:qjwang@ntu.edu.sg)


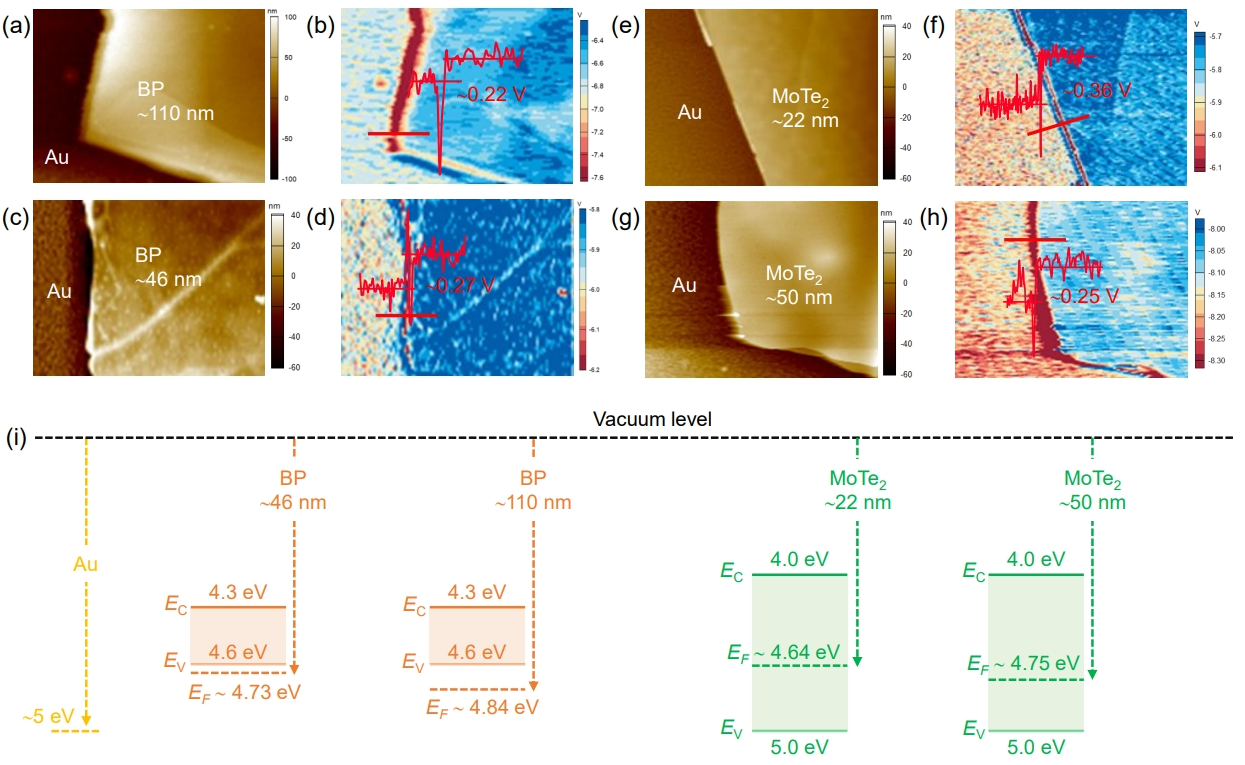


Fig. S1. KPFM characterizations of BP and MoTe_2_ with different thicknesses. (a-d) AFM and KPFM images of BP flakes on an Au substrate. (e-h) AFM and KPFM images of MoTe_2_ flakes on an Au substrate. (i) Energy band profiles of Au, BP, and MoTe_2_ with different thicknesses.

For KPFM characterization, BP and MoTe_2_ flakes with various thicknesses were transferred onto an Au substrate with a work function of approximately 5 eV. The determination of the work functions of BP and MoTe_2_ involved measuring the surface potential difference between them and the Au substrate. As shown in Fig. S1, the work function of Au was found to be higher than that of BP and MoTe_2_ flakes with different thicknesses. The surface potential difference between Au and BP flakes with thicknesses of 46 nm and 110 nm was measured at 0.27 eV and 0.22 eV, respectively, which is consistent with reported values.^1^ Similarly, the surface potential difference between Au and MoTe_2_ flakes with thicknesses of 22 nm and 50 nm was measured at 0.36 eV and 0.25 eV, respectively. Consequently, the work function of BP exhibited a slight variation from 4.73 eV to 4.84 eV with increasing thickness from 46 nm to 110 nm. In contrast, the work function of MoTe_2_ increased from 4.64 eV to 4.75 eV as the thickness increased from 22 nm to 50 nm. The minimum conduction band and maximum valence of BP and MoTe_2_ were extracted from previous reportes.^2-4^





Fig. S2. Band diagrams of unipolar and bipolar p-B-p barrier heterostructures. (a) Unipolar barrier heterostructure with zero-offset conduction bands and a large valence band barrier. (b) Bipolar barrier heterostructure with a valence band barrier and a conduction band barrier.

In infrared photodetectors, narrow-bandgap materials such as Type II InAs/GaSb superlattices, mercury cadmium telluride (HgCdTe), BP and b-AsP, are usually used as the contact and absorption layers in the unipolar barrier heterostructures.^5-12^ Taking the p-B-p unipolar barrier structure as an example, although the majority carriers can be blocked by a larger valence band barrier in a unipolar barrier p-B-p heterostructure, the free-flowing minority carriers triggered by both thermal excitation and external bias could result in substantial dark currents.^5,6^ Therefore, cryogenic cooling is required to further suppress the dark current, which is common in traditional unipolar barrier heterostructures.^13^ A proposed alternative is the bipolar barrier heterostructure, where a bipolar barrier in both valence and conduction bands can block both majority holes and minority electrons from the p-type contact and absorption layers. This design facilitates achieving lower dark currents. Additionally, the bipolar barrier layer with appropriate thickness can serve as a tunneling layer, enabling photocarriers to traverse the barrier via tunneling mechanisms. Even though the photocurrent is also suppressed by the bipolar layer, the photocurrent under bias operation could be much slower than the reduction of dark current due to reduced barrier heights under illumination and enhanced optical absorption by optical optimization, thus leading to an excellent specific detectivity. This approach may offer a promising prospect for improving the performance of mid-wave infrared photodetectors at room temperature.


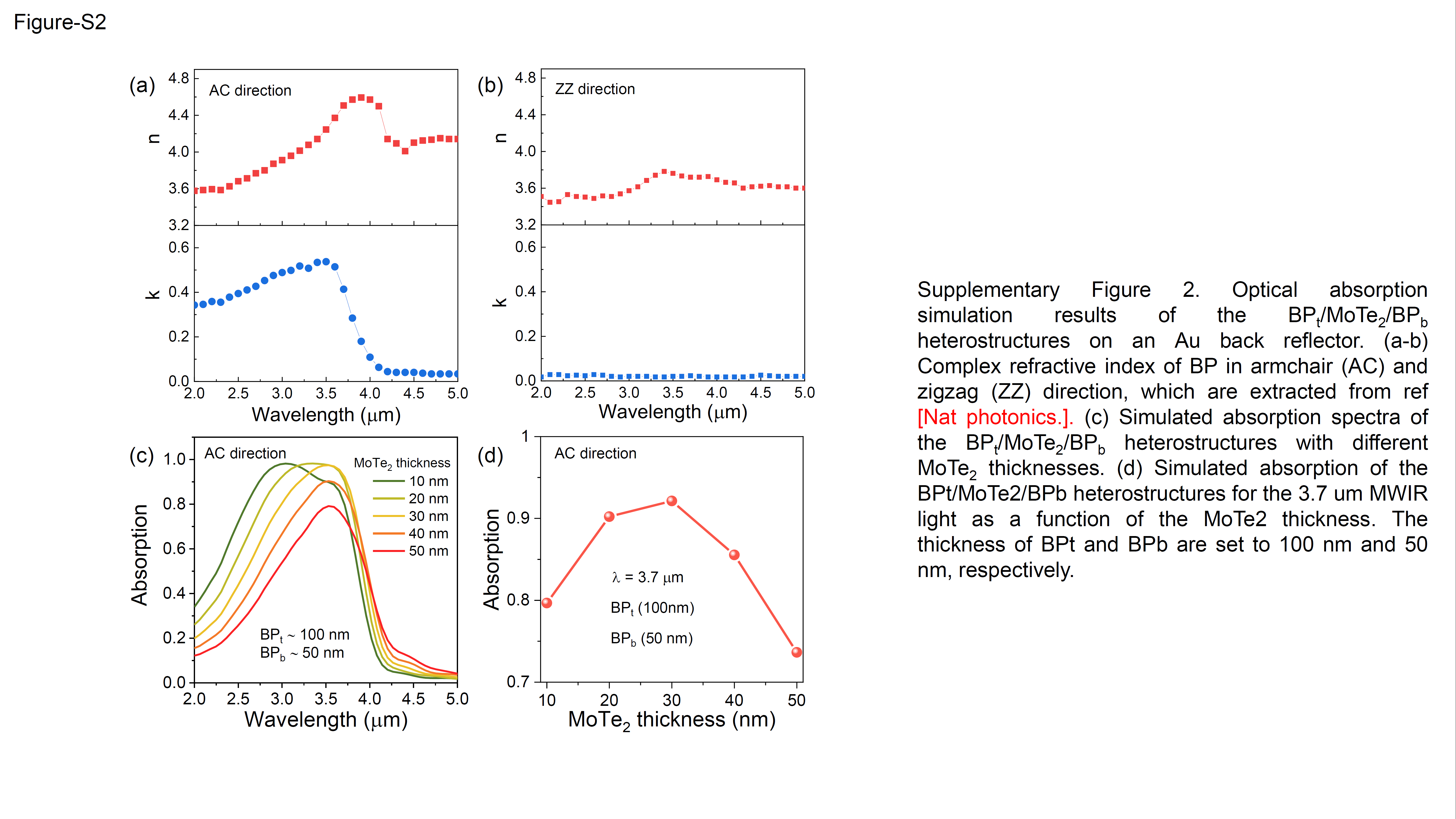


Fig. S3. Complex refractive index of BP in (a) armchair (AC) and (b) zigzag (ZZ) direction, which are extracted from the previous work [*Nat. Photon.* **12**, 601-607, (2018)].





Fig. S4. Optical absorption simulation results of the BP_t_/MoTe_2_/BP_b_ heterostructures. (a) Simulated optical absorption spectra of the BP_t_/MoTe_2_/BP_b_ (100/20/50 nm) heterostructure with and without an Au reflector. (b) Optical absorption enhancement factor at different wavelengths. (c) Simulated absorption spectra of the heterostructures with different MoTe_2_ thicknesses. (d) Simulated absorption of the heterostructures for the 3.7 μm MWIR light as a function of the MoTe_2_ thickness. The thickness of BP_t_ and BP_b_ are set as 100 nm and 50 nm, respectively.


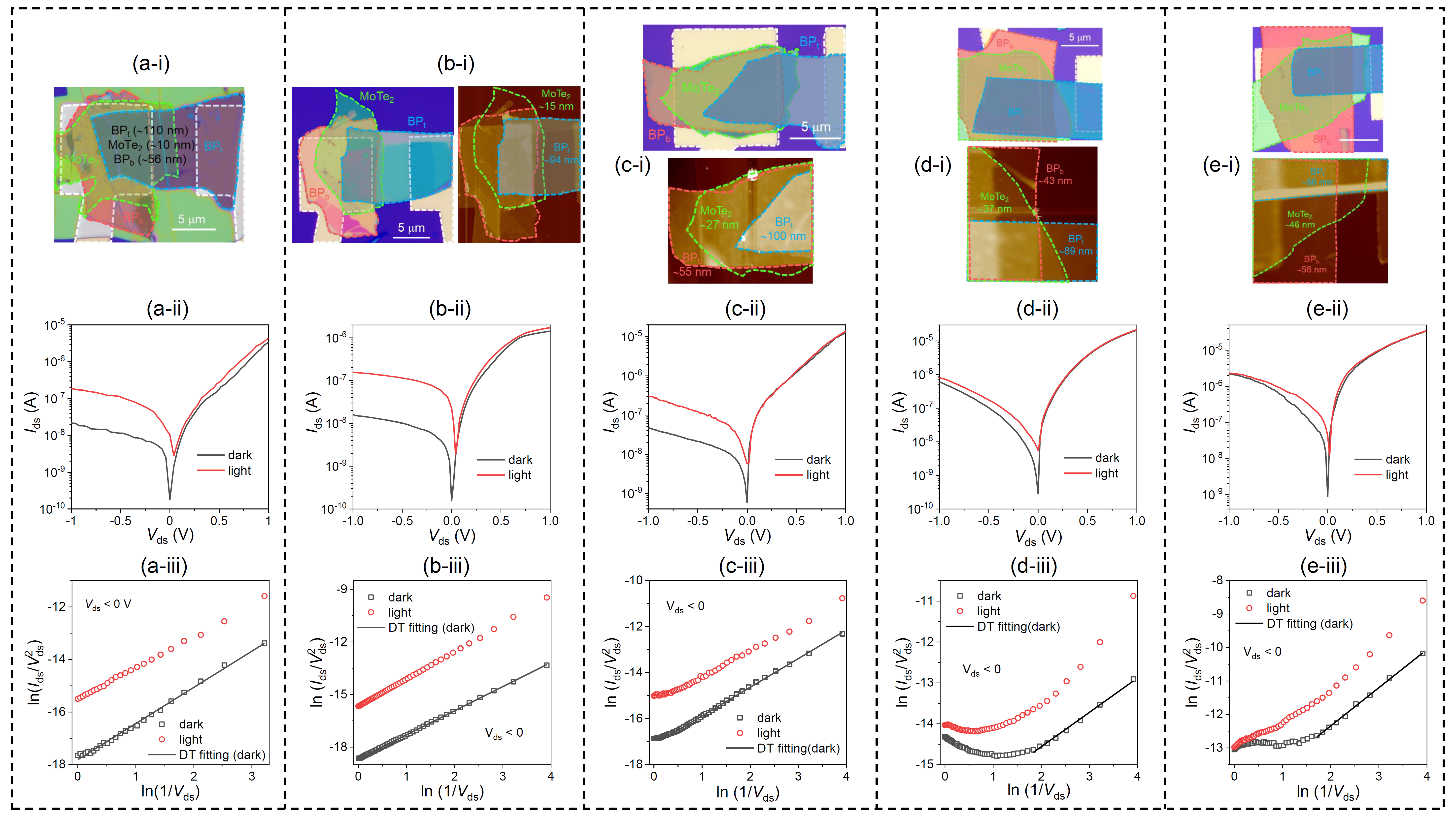


Fig. S5. The effect of the barrier layer thickness on photodetection performance. (a-i)-(e-i) Optical images and AFM images of the BP_t_/MoTe_2_/BP_b_ heterostructures. All heterostructures were finally encapsulated with h-BN flakes. (a-ii)-(e-ii) *I*_ds_-*V*_ds_ curves of the heterostructures in the dark and under 3.7 μm laser illumination with a power density of 0.05 W cm^-2^. (a-iii)-(e-iii) Direct tunneling plots of the currents at negative bias.





Fig. S6. The effect of the barrier layer thickness on photodetection dark current and photocurrent.


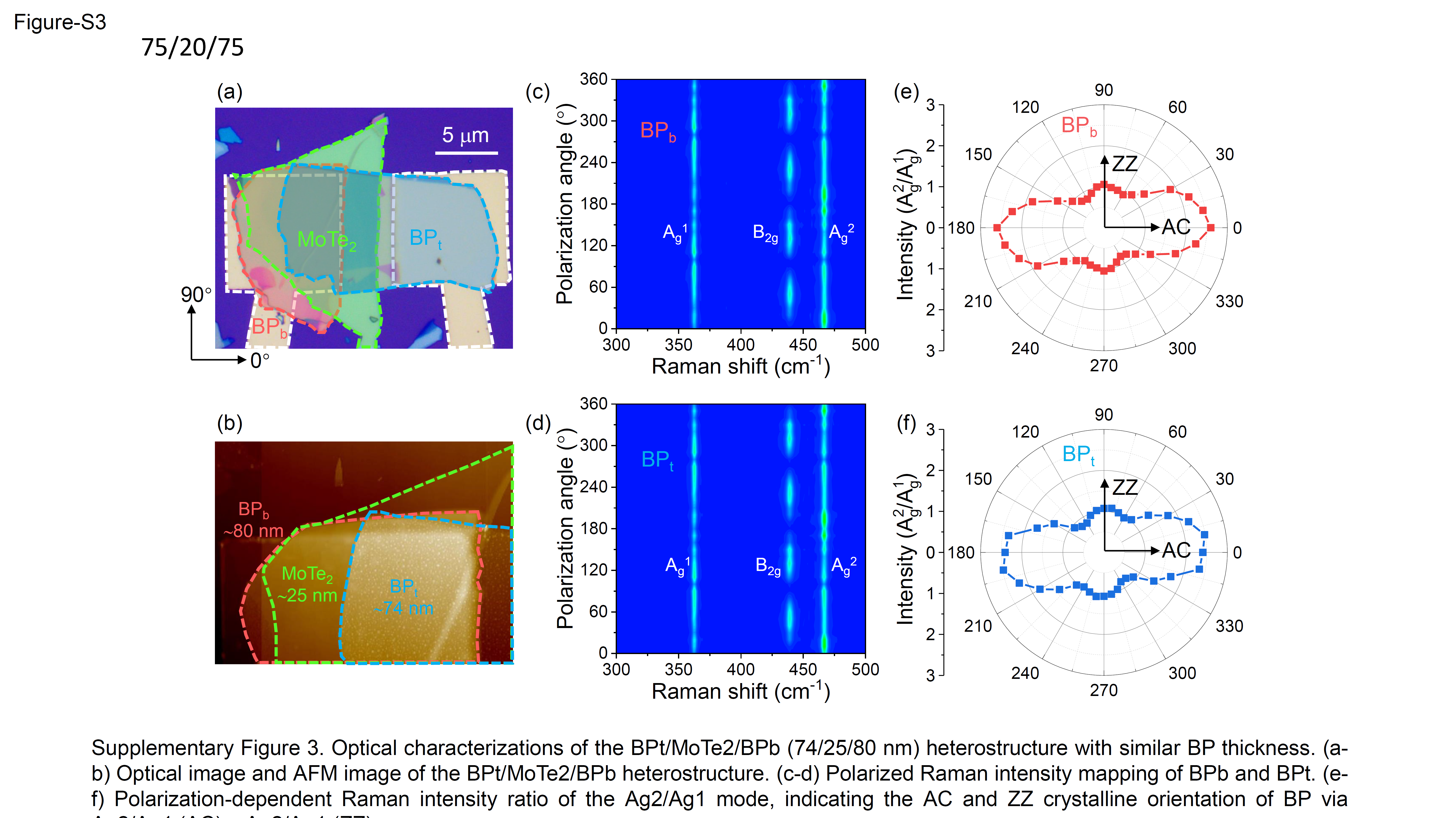


Fig. S7. Optical characterizations of the BP_t_/MoTe_2_/BP_b_ (~74/25/80 nm) heterostructure with similar BP thickness. (a-b) Optical image and AFM image of the BP_t_/MoTe_2_/BP_b_ heterostructure. (c-d) Polarized Raman intensity mapping of BP_b_ and BP_t_. (e-f) Polarization-dependent Raman intensity ratio of the A_g_^2^/A_g_^1^ mode, indicating the AC and ZZ crystalline orientation of BP via A_g_^2^/A_g_^1^ (AC) > A_g_^2^/A_g_^1^ (ZZ).





Fig. S8. Raman spectrum of the top BP layer, insert shows the morphological characterization of the BP_t_/MoTe_2_/BP_b_ heterostructure.

As presented in Fig. S8, AFM morphological characterization reveals the presence of high-density, small particles uniformly distributed across the BP surface. Raman spectroscopy analysis of these particles identifies three distinct peaks at 360 cm^-1^, 440 cm^-1^, and 467 cm^-1^, corresponding to the A_g_^1^, B_2g_, and A_g_^2^ vibrational modes of BP, respectively. A weaker peak at 390 cm^-1^, attributed to the P-O bond, is also observed, indicating the formation of phosphorus oxide (PO_x_) species.^14,15^ This suggests that the BP surface undergoes oxidation through interactions with atmospheric oxygen and moisture, leading to the formation of PO_x_ layer.


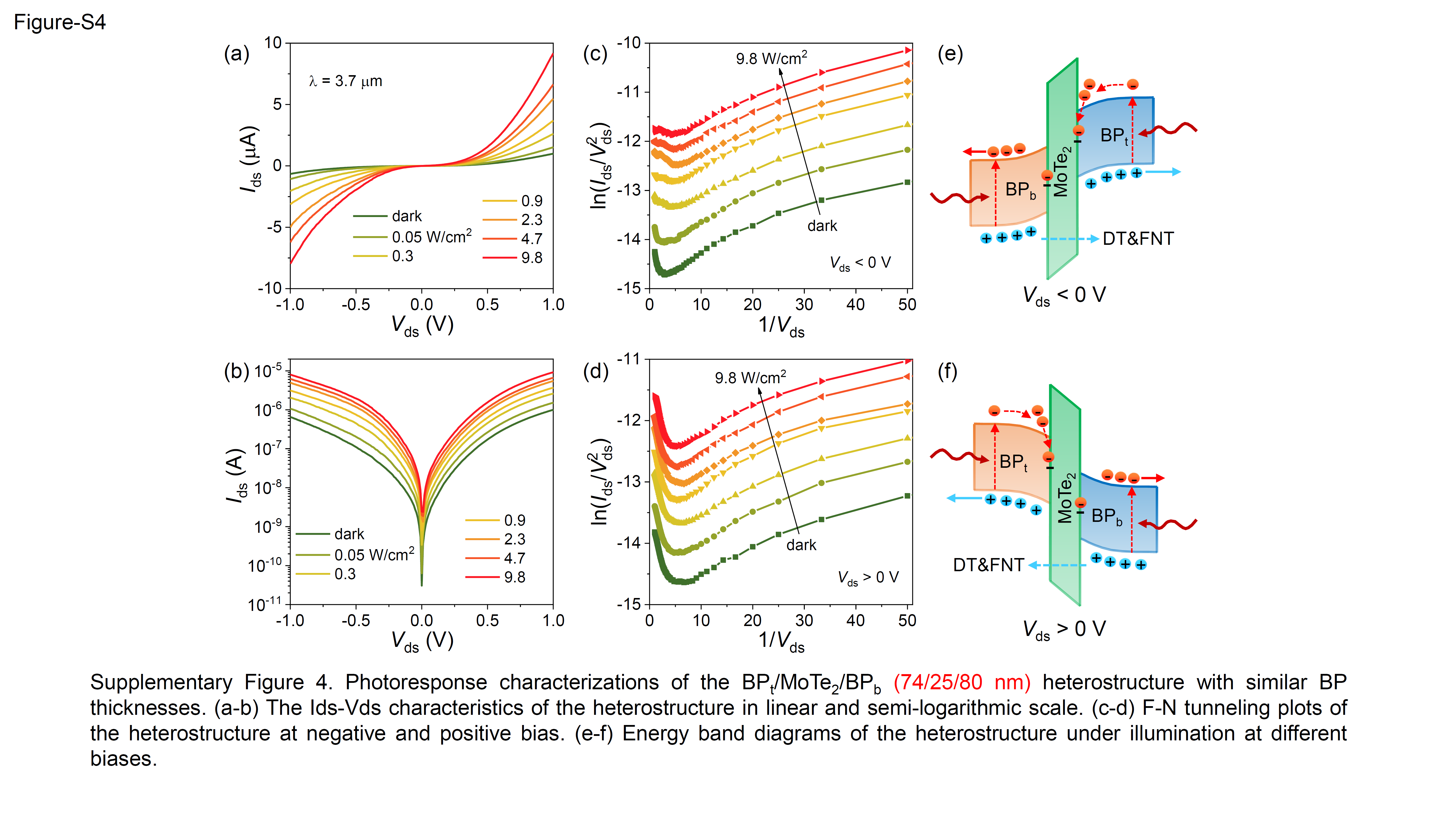


Fig. S9. Photoresponse characterizations of the BP_t_/MoTe_2_/BP_b_ (~74/25/80 nm) heterostructure with similar BP thicknesses. (a-b) The *I*_ds_-*V*_ds_ characteristics of the heterostructure in linear and semi-logarithmic scale. (c-d) FN tunneling plots of the heterostructure at negative and positive bias. (e-f) Energy band diagrams of the heterostructure under illumination at different biases.


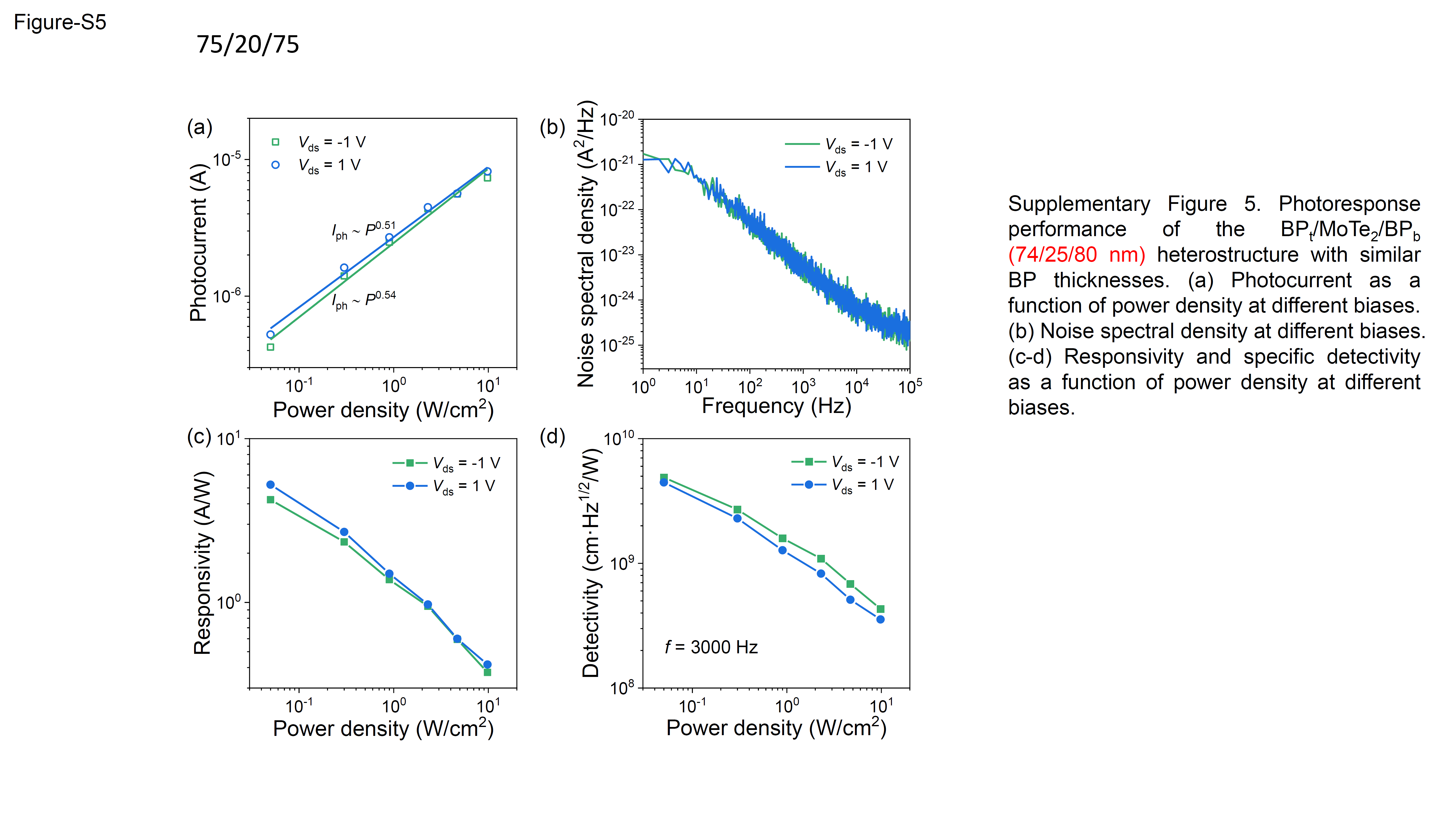


Fig. S10. Photoresponse performance of the BP_t_/MoTe_2_/BP_b_ (~74/25/80 nm) heterostructure with similar BP thicknesses. (a) Photocurrent as a function of power density at different biases. (b) Noise spectral density at different biases. (c-d) Responsivity and specific detectivity as a function of power density at different biases.





Fig. S11. Optical characterizations of the BP_t_/MoTe_2_/BP_b_ (~103/22/45 nm) heterostructure at different temperatures. (a) Measured optical absorption of the heterostructure on an Au reflector. The inset shows the optical image of the BP_t_/MoTe_2_/BP_b_ heterostructure encapsulated with a thick h-BN flake. (b) An AFM image of the heterostructure. (c-d) Polarized Raman intensity mapping of BP_b_ and BP_t_. (e-f) Raman intensity ratio of the A_g_^2^/A_g_^1^ mode at different polarization angles. The AC and ZZ crystalline orientation of BP is determined by A_g_^2^/A_g_^1^ (AC) > A_g_^2^/A_g_^1^ (ZZ).


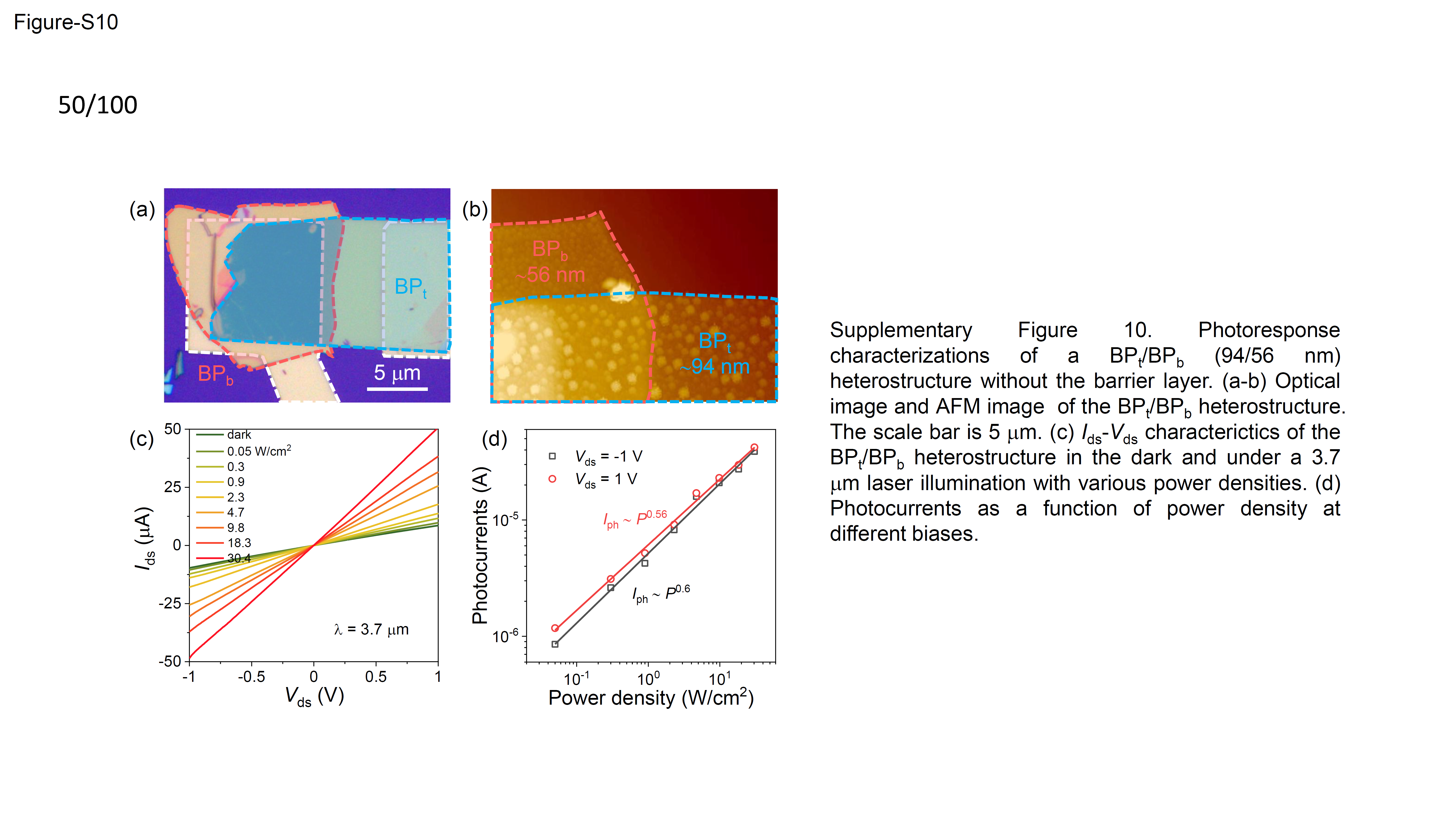


Fig. S12. Photoresponse characterizations of a BP_t_/BP_b_ (~94/56 nm) heterostructure without the barrier layer. (a-b) Optical image and AFM image of the BP_t_/BP_b_ heterostructure. (c) *I*_ds_-*V*_ds_ characteristics of the BP_t_/BP_b_ heterostructure in the dark and under a 3.7 μm laser illumination with various power densities. (d) Photocurrent as a function of power density at different biases.





Fig. S13. Temperature-dependent carrier transport of the BP_t_/MoTe_2_/BP_b_ (~103/22/45 nm) heterostructure. (a) *I*_ds_-*V*_ds_ curves of the heterostructure at different temperatures. (b-c) Arrhenius plots of dark current under (b) negative and (c) positive biases.





Fig. S14. Thermionic emission analysis of the of the BP_t_/MoTe_2_/BP_b_ (~103/22/45 nm) heterostructure. (a) Current density at different bias voltages. (b) Extracted ideality factor using the thermionic emission theory.





Fig. S15. Tunneling characterizations of the BP_t_/MoTe_2_/BP_b_ (~103/22/45 nm) heterostructure at different temperatures. (a) Direct tunneling plots of the heterostructure in the negative bias. (b) FN tunneling plots in the positive bias regime.


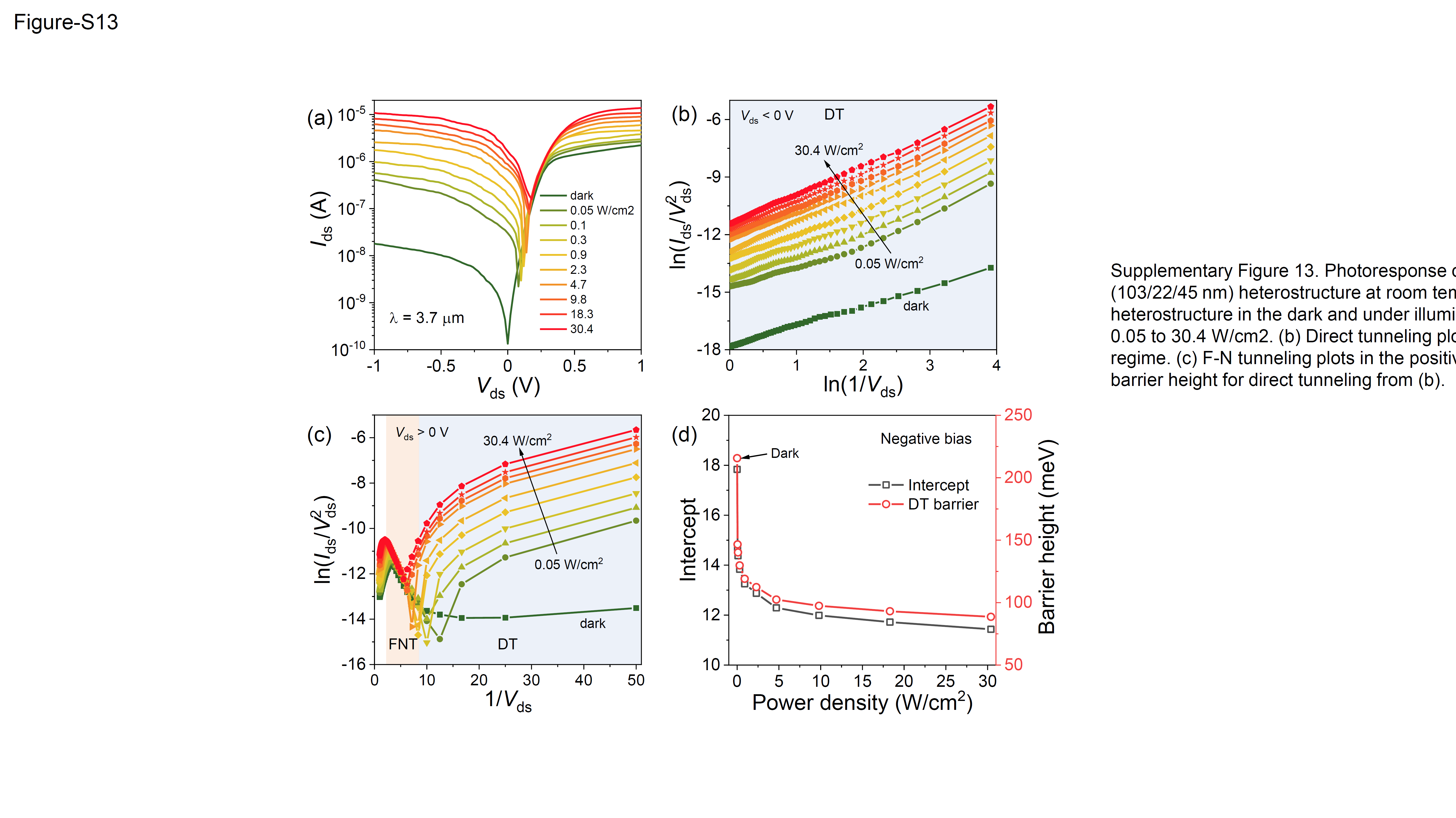


Fig. S16. Photoresponse of the BP_t_/MoTe_2_/BP_b_ (~103/22/45 nm) heterostructure at room temperature. (a) *I*_ds_-*V*_ds_ characteristics of the heterostructure in the dark and under illumination with various power densities from 0.05 to 30.4 W cm^-2^. (b) Direct tunneling plots of the heterostructure in the negative regime. (c) FN tunneling plots in the positive bias regime. (d) Extracted intercept and barrier height for direct tunneling from (b).





Fig. S17. Time-resolved photoresponse of the BP_t_/MoTe_2_/BP_b_ (~103/22/45 nm) heterostructure under different light intensities and bias voltages. (a) Photoresponse under negative bias. (b) Photoresponse under positive bias.





Fig. S18. Response dynamics of the BP_t_/MoTe_2_/BP_b_ (~103/22/45 nm) heterostructure under different light intensities and bias voltages: (a) *V*_ds_ = -1 V and *P* = 30.4 W cm^-2^. (b) *V*_ds_ = -1 V and *P* = 0.05 W cm^-2^. (d) *V*_ds_ = 1 V and *P* = 30.4 W cm^-2^. (e) *V*_ds_ = 1 V and *P* = 30.4 W cm^-2^. (c) and (f) show the response time as a function of light intensity at *V*_ds_ = -1 V and 1 V, respectively.


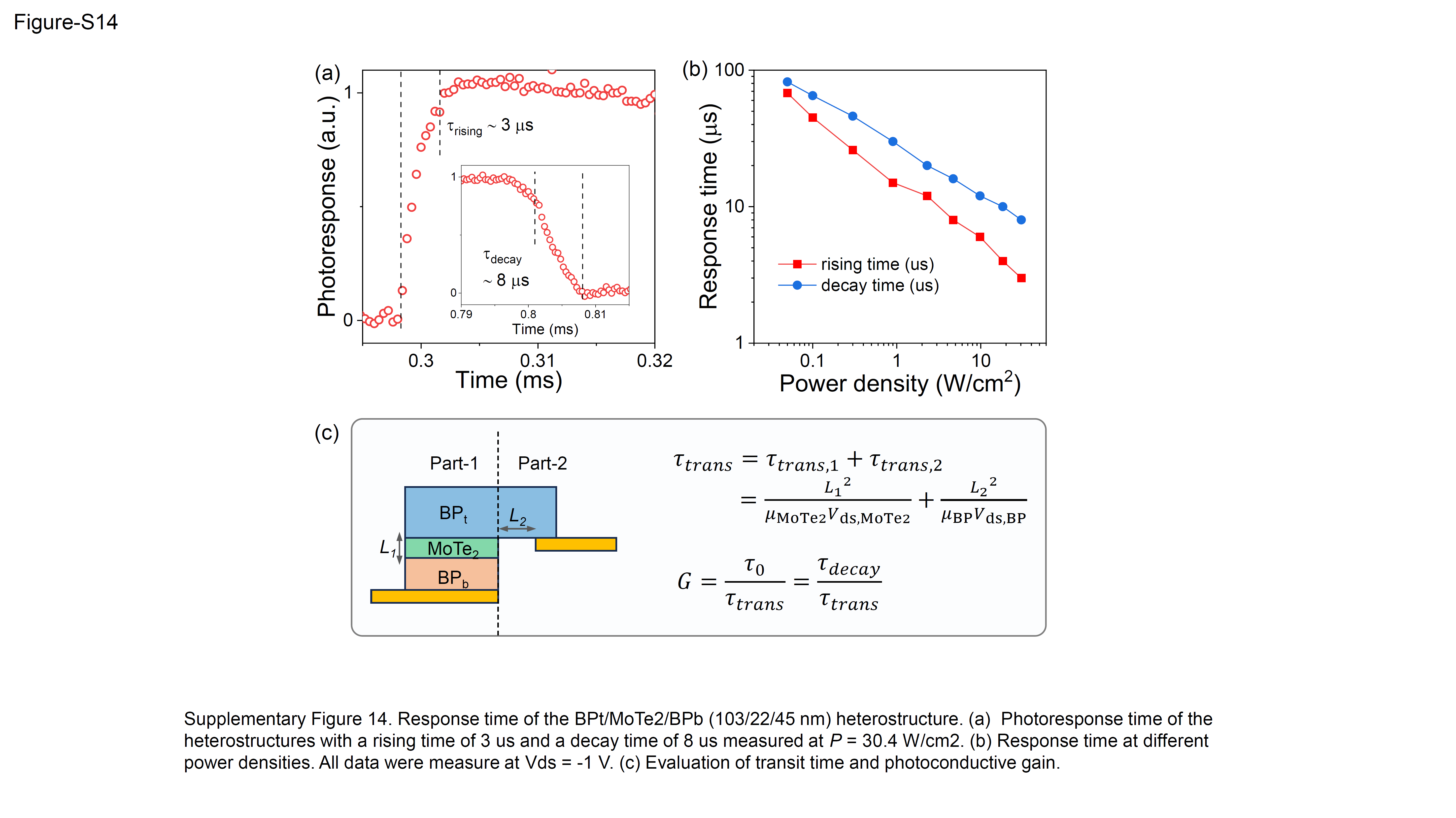


Fig. S19. Estimation methods for carrier transit time and photoconductive gain.

The carrier transit time τ_trans_ can be estimated using the formula:

τ_trans_ = *L*^2^/*μV*_ds_

where *L* is the transit distance, *μ* is the carrier mobility, and *V*_ds_ is the applied voltage. In our study, the fabricated BP_t_/MoTe_2_/BP_b_ heterostructure can be divided into two regions, as shown in Fig. S19. The region to the left of the dashed line, vertically stacked on the Au reflector, is referred to as part-1, while the right side of the dashed line, consisting of BP_t_, is part-2. Therefore, the total carrier transit time is the sum of the transit times in these two regions.

For part-1, the carrier transit time is dominated by the MoTe₂ barrier layer, as the voltage drop (*V*_ds_ = -1 V) primarily occurs across it. The transit time for carriers in part-1 can be estimated using:

τ_trans,1_ = *L*_1_^2^/*μ*_MoTe2_*V*_ds,MoTe2_

where *L*_1_ is the thickness of MoTe_2_ barrier layer (~22 nm), *μ*_MoTe2_ is the out-of-plane carrier mobility of MoTe_2_, and *V*_ds,MoTe2_ is the voltage drop across the MoTe_2_ barrier layer (1 V). According to the previous report, the out-of-plane mobility of MoTe_2_ is estimated to be 0.01 cm^2^ V^-1^ s^-1^.^16^ Thus, τ_trans,1_ is calculated to be 0.5 ns.

Similarly, the carrier transit time in part-2 is given by:

τ_trans,2_ = *L*_2_^2^/*μ*_BP_*V*_ds,BP_

where *L*_2_ is the distance between the electrodes (~5 μm), *μ*_BP_ is the in-plane mobility of BP, and *V*_ds,BP_ is the voltage drop across the BP_t_ region. From our previous work, the in-plane mobility of BP is estimated to be ~600 cm^2^ V^-1^ s^-1^.^17^ The voltage drop across the BP_t_ is assumed to be ~1 mV. As shown in Supplementary Figs. S12 and S16, the resistance of the BP_t_/MoTe₂/BP_b_ heterostructure at -1 V bias is approximately 500 times greater than that of the BP_b_/BP_t_ heterostructure. Therefore, we estimate that the voltage drops across BP_b_ and BP_t_ is around 1 mV. Based on this, the transit time in part-2 is estimated to be ~420 ns.

Thus, the overall carrier transit time is predominantly determined by τ_trans,2_. Finally, the photoconductive gain (*G*) is calculated using the formula *G* = τ_0_/τ_trans,2_, where τ_0_ represent the minority carrier recombination lifetime. The recombination lifetime τ_0_ of photogenerated minority carriers is considered equivalent to the photoresponse decay time.^18^


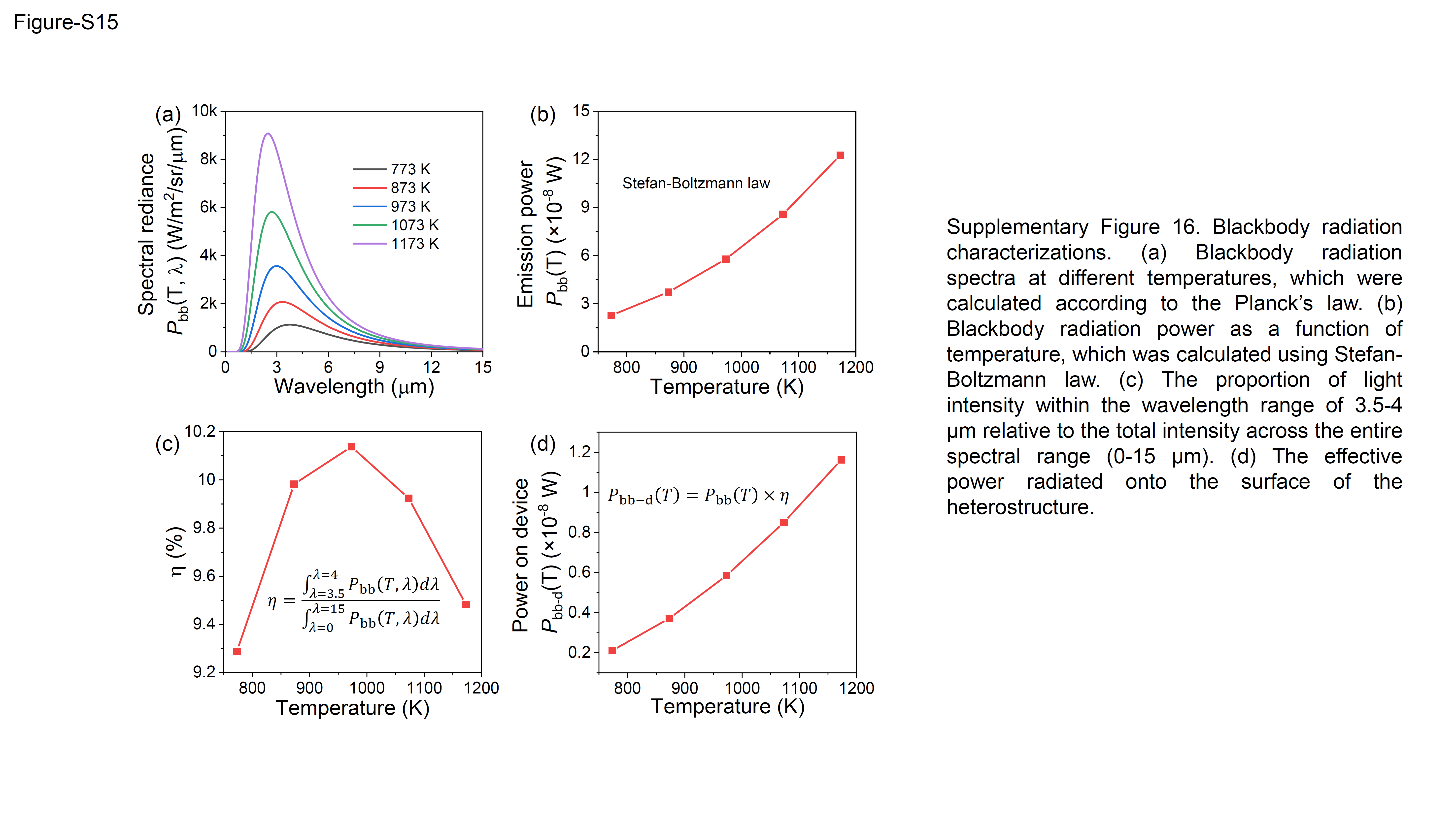


Fig. S20. Blackbody radiation characterizations. (a) Blackbody radiation spectra at different temperatures, which were calculated according to the Planck’s law. (b) Blackbody radiation power as a function of temperature, which was calculated using Stefan-Boltzmann law. (c) The proportion of light intensity within the wavelength range of 3.5-4.0 µm relative to the total intensity across the entire spectral range (0-15 µm). (d) The effective power radiated onto the surface of the heterostructure.

References

1. Xiong Y*, et al.* Twisted black phosphorus-based van der Waals stacks for fiber-integrated polarimeters. *Science Advances* **8**, eabo0375 (2022).

2. Hu S*, et al.* Gate‐switchable photovoltaic effect in BP/MoTe_2_ van der Waals heterojunctions for self‐driven logic optoelectronics. *Advanced Optical Materials* **9**, 2001802 (2020).

3. Tian R*, et al.* Chip-integrated van der Waals PN heterojunction photodetector with low dark current and high responsivity. *Light: Science & Applications* **11**, 101 (2022).

4. Zubair M*, et al.* Gate-tunable van der Waals photodiodes with an ultrahigh peak-to-valley current ratio. *Small* **19**, e2300010 (2023).

5. Chen Y*, et al.* Unipolar barrier photodetectors based on van der Waals heterostructures. *Nature Electronics* **4**, 357-363 (2021).

6. Zhang S*, et al.* Black arsenic phosphorus mid-wave infrared barrier detector with high detectivity at room temperature. *Advanced Materials* **36**, 2313134 (2024).

7. Zhang S*, et al.* Multi-dimensional optical information acquisition based on a misaligned unipolar barrier photodetector. *Nature Communications* **15**, 7071 (2024).

8. Delli E*, et al.* Mid-infrared InAs/InAsSb superlattice nBn photodetector monolithically integrated onto silicon. *ACS Photonics* **6**, 538-544 (2019).

9. Nguyen BM, Chen G, Hoang AM, Abdollahi Pour S, Bogdanov S, Razeghi M. Effect of contact doping in superlattice-based minority carrier unipolar detectors. *Applied Physics Letters* **99**, 033501 (2011).

10. Plis EA, Krishna SS, Gautam N, Myers S, Krishna S. Bias Switchable Dual-Band InAs/GaSb Superlattice Detector With pBp Architecture. *IEEE Photonics J.* **3**, 234-240 (2011).

11. He J*, et al.* Enhanced Performance of HgCdTe Long-Wavelength Infrared Photodetectors With nBn Design. *IEEE Transactions on Electron Devices* **67**, 2001-2007 (2020).

12. Kazemi A*, et al.* Mid-wavelength infrared unipolar nBp superlattice photodetector. *Infrared Physics & Technology* **88**, 114-118 (2018).

13. P. Martyniuk MK, A. Rogalski. Barrier infrared detectors. *Opto-Electronics Review* **22**, 127-146 (2014).

14. Tian B*, et al.* Facile bottom-up synthesis of partially oxidized black phosphorus nanosheets as metal-free photocatalyst for hydrogen evolution. *Proceedings of the National Academy of Sciences* **115**, 4345-4350 (2018).

15. Nan H*, et al.* Effect of the surface oxide layer on the stability of black phosphorus. *Applied Surface Science* **537**, 147850 (2021).

16. Massicotte M*, et al.* Picosecond photoresponse in van der Waals heterostructures. *Nature Nanotechnology* **11**, 42-46 (2016).

17. Wang F*, et al.* Multidimensional detection enabled by twisted black arsenic-phosphorus homojunctions. *Nature Nanotechnology* **19**, 455-462 (2024).

18. Liu M*, et al.* Photogating-assisted tunneling boosts the responsivity and speed of heterogeneous WSe_2_/Ta_2_NiSe_5_ photodetectors. *Nature Communications* **15**, 141 (2024).
